# Multilevel analysis of bacterial counts from chronic periodontitis after root planing/scaling, surgery, and systemic and local antibiotics: 2-year results

**DOI:** 10.3402/jom.v5i0.20939

**Published:** 2013-07-09

**Authors:** Ibrahimu Mdala, Ingar Olsen, Anne D. Haffajee, Sigmund S. Socransky, Birgitte Freiesleben de Blasio, Magne Thoresen

**Affiliations:** 1 Faculty of Dentistry, Department of Oral Biology, University of Oslo, Oslo, Norway; 2 Department of Periodontology, The Forsyth Institute, Cambridge, MA, USA; 3 Department of Biostatistics, Institute of Basic Medical Sciences, University of Oslo, Oslo, Norway; 4 Department of Infectious Disease Epidemiology, Divison of Infectious Disease Surveillance, Norwegian Institute of Public Health, Oslo, Norway

**Keywords:** microbiota, chronic periodontitis, periodontal therapy, antibiotics, multilevel analysis, NB GEE, fractional response methods

## Abstract

**Aim:**

To follow changes (over 2 years) in subgingival bacterial counts of five microbial complexes including health-related *Actinomyces* spp. in deeper pockets (≥5 mm) after periodontal treatments.

**Methods:**

Eight different treatments were studied: (1) scaling+root planing (SRP); (2) periodontal surgery (SURG)+systemic amoxicillin (AMOX)+systemic metronidazole (MET); (3) SURG+locally delivered tetracycline (TET); (4) SURG; (5) AMOX+MET+TET; (6) AMOX+MET; (7) TET; and (8) SURG+AMOX+MET+TET. Antibiotics were given immediately following SRP. Subgingival plaque was collected mesiobuccally from each tooth, except third molars, from 176 subjects, completing the study, at baseline, 3, 6, 12, 18, and 24 months post-treatment and analysed for 40 different bacteria using checkerboard hybridization. A negative binomial (NB) generalized estimating equation (NB GEE) model was used to analyze count data and a logistic GEE was used for proportions.

**Results:**

We observed short-term beneficial changes in the composition of the red complex of up to 3 months by treating subjects with AMOX+MET+TET. Similar short-term improvements with the same treatment were observed for *Tannerella forsythia* and *Treponema denticola* of the red complex. SURG had also short-term beneficial effect on *Porphyromonas gingivalis*. No periodontal treatments applied to severely affected sites promoted the growth of *Actinomyces*. Smoking elevated counts of both the red and orange complex while bleeding on probing (BOP) and gingival redness were also predictors of more red complex counts. Comparatively similar findings were obtained by analyzing counts and by analyzing proportions.

**Conclusions:**

Although short-term reductions in the counts of the red complex were observed in sites that were treated with AMOX+MET+TET, long-term significant effects were not observed with any of the eight treatments. Poor oral hygiene in patients with severe chronic periodontitis diminished the beneficial effects of treatment.

In a previous 2-year study on the clinical effects of periodontal treatments (1), we found that combining periodontal surgery (SURG) with systemic amoxicillin (AMOX) and systemic metronidazole (MET), and locally delivered tetracycline (TET) produced significant clinical improvement of chronic periodontitis. It was concluded that both surgical and non-surgical therapies can be used to arrest chronic periodontitis. SURG+AMOX+MET+TET gave the best maintenance of clinical results over a 2-year period. Site-level effects such as bleeding on probing (BOP), accumulation of plaque, gingival redness, and suppuration were significant predictors of further loss of clinical attachment level (CAL) and increased pocket depth (PD). In this study, we report the effects of these treatments on subgingival bacteria.

Studies have demonstrated that five different clusters or communities of bacteria may be found in subgingival plaque of subjects with chronic periodontitis. The red complex consisting of *Tannerella forsythia*, *Porphyromonas gingivalis*, and *Treponema denticola* is frequently found in deeper periodontal pockets and its presence is usually preceded by species of the more diverse orange complex. The red complex is the one most related to periodontitis clinically. In contrast, species of the yellow, green, and purple complexes are considered to be host compatible and are generally associated with healthy sites. Thus, chronic periodontitis has a polymicrobial etiology (2).

Systemic or locally applied antibiotics have been used for years in the treatment of periodontitis. The idea of using antibiotics in the treatment is that they should lead to beneficial changes in the composition of the subgingival microbiota by reducing pathogens and allowing the growth of host-compatible species (3). Previous studies have shown that systemic treatment with MET, which act against anaerobic bacteria, has beneficial effects on the subgingival microbiota of periodontal diseases (4). Fluctuation or at least quantitative changes in the incidence of potential periodontal pathogens were reported after administration of MET (5). MET has also been compared directly to other antibacterial substances in the treatment of periodontitis. All such treatments, including MET, azithromycin, sub-antimicrobial doses of doxycycline, and scaling and root planing (SRP), reduced counts of red complex species at 12 months but no significant differences were detected among treatment groups for most species (6). The greatest benefits in clinical and microbiological parameters in smokers with chronic periodontitis were achieved with the use of SRP+MET+AMOX (7) and systemic administration of AMOX+MET or MET had a significant effect on the levels of important periodontal pathogens for 6 months post-therapy (8). Systemic use of doxycycline or MET+AMOX led to levels of *Aggregatibacter actinomycetemcomitans* below detection limits in localized juvenile periodontitis at 10, 30, and 90 days post-treatment (9). In smokers, adjunctive local doxycycline caused greater reduction in the frequency of *P. gingivalis* after initial and supportive therapy compared to conventional treatment (10). Two local drug delivery systems, one containing MET, the other TET hydrochloride, gave both improvements in microbiological parameters of chronic periodontitis (11). The improvement was greater when the local antibiotic therapies were used as an adjunct to mechanotherapy and the results were sustained 90 days following therapy. Both AMOX and MET gave significant decreases in counts of many of the bacterial species associated with periodontitis, particularly in the red and orange complexes (12). Reduced red complex species were maintained up to 12 months, especially in patients treated with MET. Mestrik et al. (13) found that the AMOX+MET group had the lowest proportions of the red complex as well as a significant decrease in the proportions of the orange complex bacteria after treatment. Other authors have reported that the combination of MET+AMOX is effective in combating *A. actinomycetemcomitans*- and *P. gingivalis*-associated periodontal infections (14–16). Cionca et al. (17) reported excellent clinical results in the antibiotic group (AMOX+MET) regardless of the presence or absence of six classic periodontal pathogens prior to treatment. Also in a short-term study, Buchmann et al. (18) found that AMOX+MET accelerated the suppression of the periodontal microbiota compared to non-surgical therapy (SRP), but had limited effect on the elimination of target isolates during healing. Valenza et al. (19) reported a favorable outcome after mechanical plaque removal and systemic AMOX+MET, but only a transient effect on *P. gingivalis* and *T. denticola* phylotypes. *T. socranskii*-like phylotypes were not affected. Furthermore, Ribeiro et al. (20) found no improvement in the microbiologic and immunologic outcome after full-mouth debridement associated with AMOX+MET in the treatment of severe chronic periodontitis.

Control of periodontal disease may best be obtained by eliminating or suppressing the causative organisms and by establishing a host-compatible microbiota. Most studies have been too short to demonstrate this and beneficial species have rarely been examined. The number of species studied has also tended to be too limited to monitor the effect of treatment on both putative periodontal pathogens and host-compatible species. Rather than concentrating on specific organisms, recent research has indicated that examination of bacterial complexes may be more useful for addressing the question of periodontal pathogenesis and the effects of therapy. By using checkerboard DNA–DNA hybridization (21), the presence and levels of 40 cultivable periodontal species with a putative pathogenic or a host-compatible role were followed for 2 years in this study. Species defined under each of the five bacterial complexes in subgingival plaque (2) were assessed before and after different treatments. In addition, selected *Actinomyces* species, thought to be associated with periodontal health were monitored.

## Materials and methods

The microbiological data were collected from 217 subjects with chronic periodontitis at two different periodontal centres in Boston, USA and Göteborg, Sweden (22). Subgingival plaque was collected mesiobuccally from each tooth, except third molars. We considered data from 1,463 teeth with an initial PD of at least 5 mm from 176 subjects who completed the study, giving an average of 8.3 teeth per subject. The patients were monitored at baseline and at 3, 6, 12, 18, and 24 months.

The subgingival plaque was sampled separately, after first removing supragingival plaque, from each site with individual sterile Gracey curettes and assessed for the contents of 40 subgingival bacterial species using checkerboard DNA–DNA hybridizations (21). Each sample was transferred to a tube containing 0.15 ml TE (10 mM Tris-HCl, 0.1 mM EDTA, pH 7.6), with 0.15 ml freshly prepared 0.5 M NaOH added. The samples were boiled for 5 min and neutralized with 0.8 ml 5 M ammonium acetate and placed in the extended slot of a Minislot (Immunogenetics, Cambridge, MA, USA) and concentrated on a positively charged nylon membrane (Roche, Indianapolis, IN, USA) by vacuum and fixed to the membrane by exposure to ultraviolet light, succeeded by baking at 120°C for 20 min. The chemiluminescence signals from each membrane were read using a Lumilmager Workstation (Boehringer-Mannheim, Mannheim, Germany) and evaluated by comparing the signals obtained with those of pooled standard samples containing 10^6^ and 10^5^ bacteria of each of the 40 bacterial species. The probes used had been tested for specificity and cross-hybridization, as described previously (21).

The response variables were counts of *Actinomyces* and counts of the red, orange, yellow, green, and purple complexes (2). The treatment effects on individual species of the red complex were also studied. We evaluated the long-term benefits of the therapies either individually or as combinations.

The treatments evaluated were: (1) SRP alone; (2) SRP+SURG+AMOX+MET; (3) SRP+SURG+TET; (4) SRP+SURG; (5) SRP+AMOX+MET+TET; (6) SRP+AMOX+MET; (7) SRP+TET; and (8) SRP+SURG+AMOX+MET+TET. For details concerning patients, inclusion and exclusion criteria, periodontal treatments and clinical monitoring Goodson et al. (22) and Mdala et al. (1) should be consulted. In brief, exclusion criteria were known allergies to the antibiotics being used, antibiotic or periodontal treatment within the last 3 months, pregnancy, nursing, or systemic conditions that would influence the course of periodontal treatment, or allergy to AMOX, MET, TET, lidocaine, or chlorhexidine. Both males and females of all races who were at least 20 years of age, had at least 15 natural teeth, and were in good general health were included in the study. These patients had at least four teeth with pockets >5 mm and at least eight teeth with CAL >3 mm. At baseline, all subjects received SRP performed under local anaesthesia and usually finished in four weekly visits. SRP was performed by treating a quadrant at a time under local anaesthesia at approximately weekly intervals. During SRP treatment, subjects rinsed twice daily with 0.1% chlorhexidine. Systemic antibiotics were given as 14 days of medication (MET 250 mg×3 and AMOX 500 mg×2). This therapy was started immediately after the first session of SRP and was administered in parallel to local TET fibre application. Compliance was reported by the patients. Local antibiotics were given as TET fibres (Actisite^®^, Procter & Gamble, Cincinnati, OH, USA) in pockets ≥5 mm immediately after mechanical instrumentation at the SRP visits. The fibres were removed after 7 days, that is, at the next appointment for quadrant-wise SRP. Surgery was performed at the 3-month monitoring visit following SRP. Subjects who had residual PDs ≥5 mm and BOP received modified Widman flap surgery at weekly intervals as needed. Chlorhexidine, 0.1%, was used as a mouth rinse for 1 min twice daily during the surgical phase and 2 weeks following the last surgical session. Each subject was provided with a powered toothbrush and triclosan-containing toothpaste and instructed to brush twice daily and to perform daily interdental cleaning with dental floss, toothpicks, and/or interdental brushes. At each follow-up visit, the patients’ oral hygiene standard was checked and reinforced when indicated. Furthermore, at the 12-month recall, all sites with PD ≥5 mm and BOP were subjected to subgingival mechanical debridement.

## Count response models

The Poisson regression model is the basic model for modelling count data and has a probability distribution given as

$$f(y;\mu )=\frac{{\text{e}}^{-\mu_{i}}{\left(\mu_{i}\right)}^{y_{i}}}{y_{i}!},y_{i}=0,1,2,...,n_{i};\mu >0$$

where *y* is the count response random variable and the parameter *µ* is the mean of the distribution. A unique feature of this distribution is that its mean is equal to its variance, a relationship called equidispersion. However, it is very uncommon to have real data satisfying this property. In most cases, data are over-dispersed, that is, the variance is greater in value than the mean. This distributional assumption may also be violated when there are excessive zero counts. Yet another assumption behind the Poisson regression model is the requirement that counts are independent of one another. In longitudinal studies, data are in the form of panels. Observations within panels cannot be considered to be independent but may be highly correlated. In addition, we had measurements from several teeth per patient, giving rise to another level of dependence.

The distributional problems discussed above have led to development of more general count models, which allow for the modelling of a wide range of count response situations. The NB model is one of them.

### The NB model

The NB model is the traditional model of choice for modelling over-dispersed Poisson data. It is derived as a Poisson–gamma mixture distribution and is basically an extension of the Poisson model with an additional ancillary or heterogeneity parameter. Several distinct NB models have been developed to account for distributional problems associated with count data (23). A generalized NB model has a variance function with a characteristic form of *µ*+*αµ* ^*p*^. In the case of the Poisson model, this heterogeneity parameter is equal to 0 (*p*=0). The traditional NB regression model (*p*=2) is termed the NB2 model (24) owing to the quadratic nature of its variance function *µ*+*αµ* ^2^. The negative binomial 1 (NB1) model has a variance function given as *µ*+*αµ* and is rightly called a linear parameterization. However, just like the Poisson model, the NB model may also be over-dispersed. Both models can be extended and modified to accommodate any extra correlation in the data that violate distributional properties of these models.

### Violations of distributional properties and extensions considered

Modelling bacterial count data is often challenging due to disproportionately high numbers of zeros in the response variable. In addition to this Poisson violation, our data were structured in panels, with repeated observations per tooth and teeth nested within patients. Zero-inflated count models, which were first introduced by Lambert (25), have the capacity to handle excessive zero counts. These models include the zero-inflated Poisson (ZIP), which is a subset of the zero-inflated negative binomial (ZINB). The only drawback with these models is their limited abilities to handle nested or clustered data from longitudinal studies. This second problem is addressed by the so-called generalized estimating equations.

### Generalized estimating equations

Generalized estimating equations (GEE) (26) is one of the most widely used statistical methods in the analysis of longitudinal data (27). It is an extension of the generalized linear model (GLM) (28) to account for possible correlations between the repeated measurements of an individual over time. GEE, which is a population averaging panel method, accounts for the correlation between observations in GLM by introducing a working correlation matrix and by using robust variance estimators. The method differs from a random effects model, which is subject-specific, by averaging the model effects across individuals. The benefit of the GEE approach is that the correlation matrix can be arbitrarily parameterized. The following correlation structures were considered for our data; independence, exchangeable, unstructured, autoregressive, stationary, and non-stationary. The exchangeable correlation structure, which had the smallest quasi-likelihood independence criterion (QIC), was chosen. This structure assumes that the correlations between all observations within a panel are the same. This implies that any correlation value within the structure may be exchanged with any other. However, as stated by Zeger and Liang (26), the GEE approach is very attractive because estimates from the GEE method are asymptotically consistent even if the correlation structure has been misspecified.

### Model description

Let *Y*=(*y*_*i*2_,…,*y*_*ij*_) be a vector of count response variables of the bacteria in any of the complexes from *i*=1,…,176 subjects with *j*=2,…,6 time visits corresponding to 3, 6, 12, 18, and 24 months, respectively. For each *y*_*ij*_, covariates *x*_*ij*_ are given. We modelled the time course from visit 2 to visit 6 and made adjustment for baseline by including the baseline counts as a covariate. As the GEE is generally restricted to one level of correlation, we considered teeth as a factor in the model with the form:

$$\text{log}\left[E\left(Y|x_{ij}\right)\right]{=\beta }_{\text{0}}+\beta_{1}{\text{time}}_{j}+\beta_{2}{\text{treat}}_{i}{+\beta }_{\text{3}}{\text{age}}_{i}{+\beta }_{\text{4}}{\text{gender}}_{i}{+\beta }_{\text{5}}{\text{nationlity}}_{i}{+\beta }_{\text{6}}{\text{smoking}}_{i}{+\beta }_{\text{7}}{\text{plaque}}_{i}{+\beta }_{\text{8}}{\text{BOP}}_{i}{+\beta }_{\text{9}}{\text{PD}}_{i}+\beta_{10}{\text{tooth}}_{i}{+\beta }_{\text{11}}{\text{AL}}_{i}{+\beta }_{\text{12}}{\text{baseline\_counts}}_{i}+\beta_{13}{\text{time}}_{j}{\text{treat}}_{i}$$

where *E*(*Y*∣*x*_ij_) is the expectation of *Y* given the covariates *x*_*ij*_. The parameter estimates β_0_ through to **β**_**13**_ represent fixed effects associated with the intercept, time effect, treatment, age, gender, nationality (either American or Swedish), smoking habits, plaque accumulation, BOP, PD, tooth, CAL, baseline counts, and the interaction of treatment with time. Here, β_0_ is the intercept, that is the expected count assuming a model with no covariates, **β**_**1**_ is a vector of four coefficients representing the effect of time treated as a factor (relative to visit 2), **β**_**2**_ is a vector of seven coefficients representing the effect of the seven treatment groups relative to the reference group SRP and **β**_**10**_ is correspondingly a vector of 28 coefficients representing tooth effect. Finally, **β**_**13**_ is a vector of 28 coefficients representing the interaction between the factor time and the factor treatment.

### Correlation structure and model selection

The Akaike information criterion (AIC) (29), which is normally used for GLM model selection is not applicable with GEE models. This is because GEE models are not based on the likelihood function (30). Pan (31) proposed the quasi-likelihood under the independence model criterion (QIC) for selecting the best model in GEE analyses. The QIC value can also be used to select the best correlation structure. The exchangeable correlation structure, which had the smallest QIC, was selected. Then, under the exchangeable correlation structure, the model with the smallest QIC value among the different models fitted with this working correlation was considered to be the best GEE model.

### Modelling fractional responses

Fractional response variables are encountered in many areas of research including biological settings. As data on proportions are bounded between 0 and 1, the use of standard linear models becomes less appealing. For example, assuming that *y* is a fractional response satisfying the relation 0≤*y*≤1, then if we implement a standard linear model, we risk predicting proportions that are less than 0 or more than 1. This argument also holds for panel data models. One is faced with the problem of imposing a positive bound effect on the proportions.

Several approaches of handling data on proportions have been suggested in the literature. The most commonly used model is the logistic regression model. We used the logistic model with GEE to handle the dependence in the data in the same way as described above for the analysis of counts. However, it should be noted that the interpretation of the two approaches differs. While the logistic GEE model looks at the extent to which the treatments influenced the total composition of the bacteria, the GEE for counts looked at the extent to which the treatments influenced the different levels of the bacteria complexes.

### Significance level

We performed a rather high number of statistical tests in this study. To reduce the multiple testing problems, we used a significance level of 1%, and correspondingly, we computed 99% confidence intervals in our tables. However, results that are significant at 5% level are also indicated, by a single asterisk.

## Results

As shown in Table 1, there were no significant baseline differences between subjects in the control group (SRP) and those receiving other treatments.

**Table 1:** Baseline characteristics of the study subjects

	Treatments*								
		
	1	2	3	4	5	6	7	8	*p*
*n*	26	19	25	22	28	26	25	25	0.44
Age (years)	50±12	54±12	54±11	54±9	53±7	50±10	50±10	55±12	0.39
Americans (*n*)	14	8	11	10	15	13	12	12	0.99
Females (*n*)	13	8	13	9	15	10	15	13	0.78
									
Smoking status – number (%)									
Current smoker	9 (35)	8 (42)	11 (44)	7 (32)	14 (50)	10 (38)	8 (32)	11 (44)	0.91
									
Mean log counts									
Red	13.7±2.4	13.5±2.9	13.2±3.1	13.7±2.6	13.4±3.2	13.7±2.7	13.6±2.1	13.6±2.1	0.55
Orange	14.7±1.7	14.7±1.8	15.0±1.5	14.6±1.9	14.5±2.0	14.8±1.9	14.7±1.9	14.9±1.5	0.41
*Actinomyces*	13.2±2.7	13.8±1.7	13.4±2.8	13.4±2.9	13.5±2.3	13.3±2.9	13.5±2.2	13.6±2.4	0.43
Yellow	12.2±2.9	10.9±3.5	11.5±3.3	11.6±3.0	11.8±3.2	11.8±3.0	12.3±2.5	12.0±2.5	0.52
Purple	12.2±3.0	12.6±2.3	12.7±2.3	12.1±3.1	12.2±3.0	11.9±3.2	12.3±2.6	12.6±2.7	0.16
Green	13.4±2.4	13.9±1.5	13.4±2.4	13.4±2.5	13.3±2.3	13.3±2.3	13.5±1.9	13.5±2.4	0.31
									
Percentage of sites with									
Plaque	23.6	37.5	26.8	32.4	336.6	27.1	32.8	37.8	
Gingival redness	33.5	34.3	33.9	32.6	38.3	33.5	27.8	40.0	
Bleeding on probing	50.0	34.9	32.6	41.9	32.3	26.2	44.4	37.2	
Mean PD (mm)	6.4±1.3	6.6±1.8	6.4±1.3	6.5±1.3	6.8±1.8	6.5±1.5	6.5±1.3	6.6±1.6	0.61
Mean CAL (mm)	6.0±2.4	5.9±2.5	6.3±2.0	6.0±1.8	6.7±2.3	6.9±2.3	6.0±1.7	6.7±2.5	0.29
Number of missing teeth	6.6±5.6	6.8±5.2	7.3±4.7	7.8±4.8	6.6±5.6	7.3±5.8	7.9±4.5	8.2±5.1	0.82

**Fig. 1:**
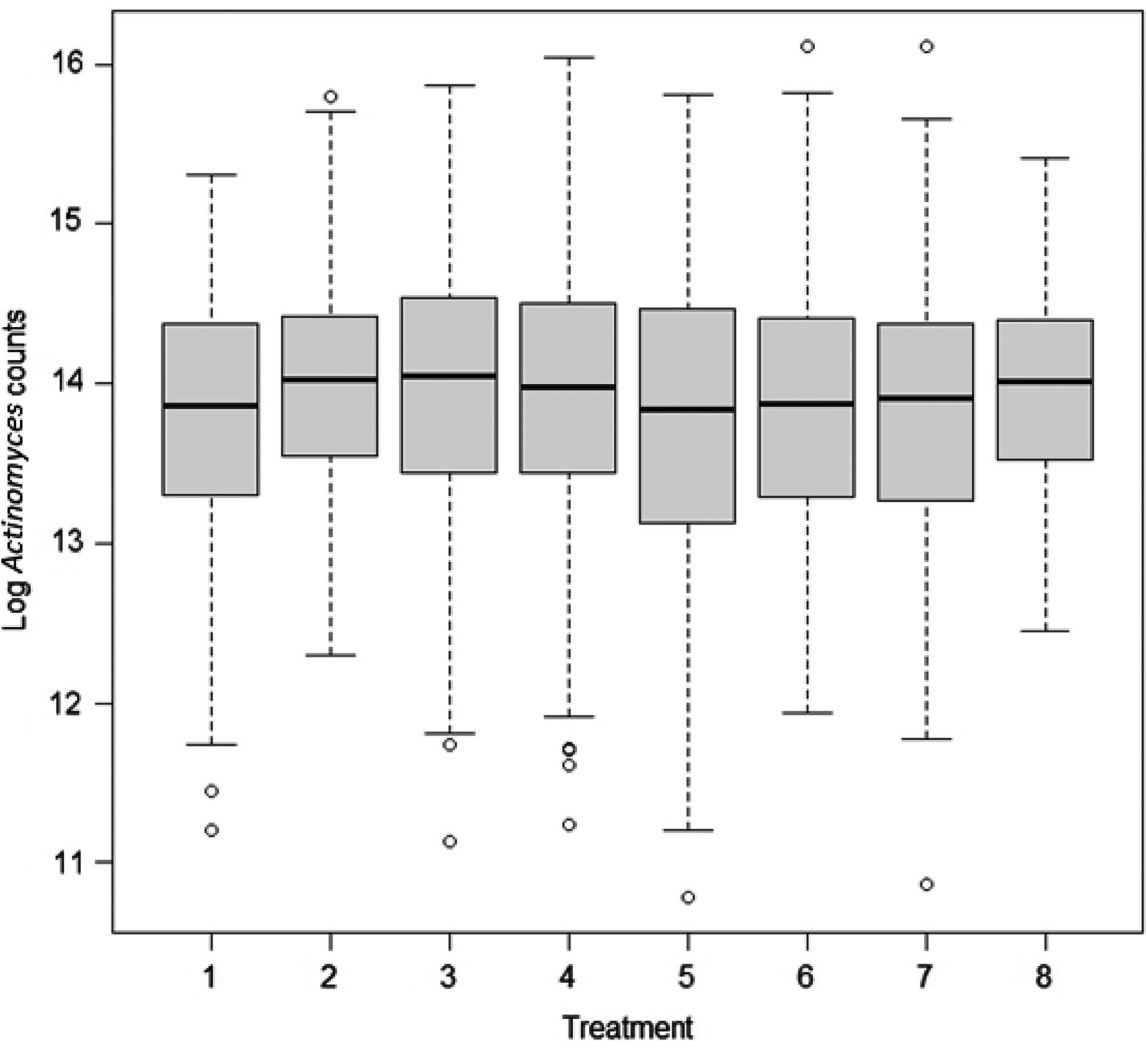
Box-plot showing the median distribution of *Actinomyces* counts by treatment group at baseline. Treatments 1–8 are SRP, surgery (SURG)+AMOX+MET, SURG+TET, SURG, AMOX+MET+TET, AMOX+MET, TET, and SURG+AMOX+MET+TET, respectively. Minimal variations in the median distributions of the counts can be seen. The circles in the plot indicate outliers.

**Fig. 2:**
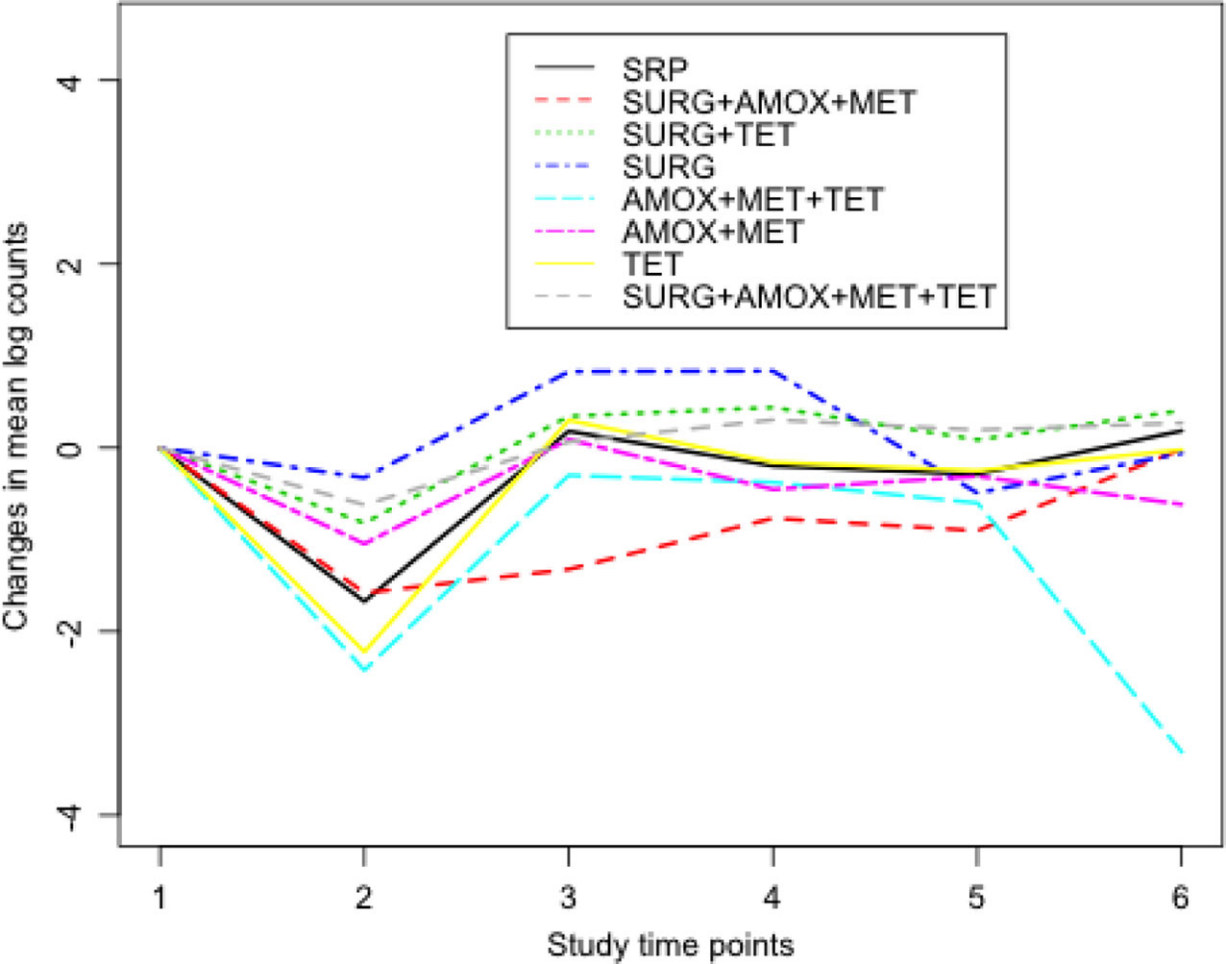
Changes in natural log counts of *Actinomyces* at each study time point from baseline. Study time points 1–6 correspond to baseline, 3, 6, 12, 18, and 24 months, respectively. Lower counts in all treatment groups can be seen at 3 months compared to baseline counts. Compared to the reference group SRP, subjects in the AMOX+MET+TET and TET groups had lesser counts between baseline and 3 months.

**Fig. 3:**
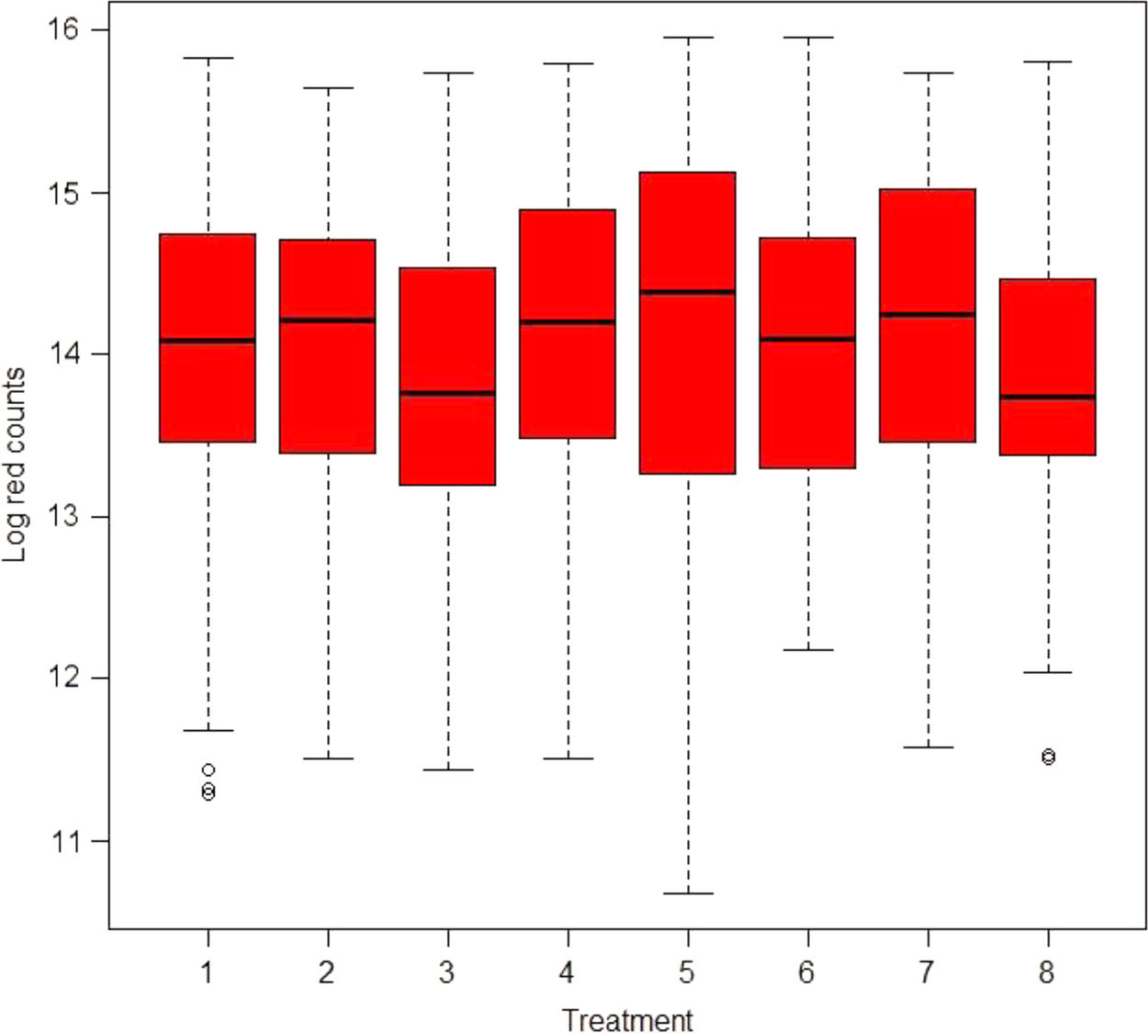
Box-plot showing the median distribution of the red complex counts by treatment at baseline. Treatments 1–8 are SRP, surgery (SURG)+AMOX+MET, SURG+TET, SURG, AMOX+MET+TET, AMOX+MET, TET, and SURG+AMOX+MET+TET, respectively. Lesser counts of the red complex were observed at baseline in the SURG+TET and SURG+AMOX+MET+TET-treated groups. The circles in the plot indicate outliers.

* The eight treatments were: 1. Scaling and root planing (SRP). 2. SRP+surgery (SURG), systemic amoxicillin (AMOX)+metronidazole (MET). 3. SRP+SURG + locally delivered tetracycline (TET). 4. SRP+SURG. 5. SRP+systemic AMOX+MET and local TET. 6. SRP+systemic AMOX+MET. 7. SRP+locally delivered TET. 8. SRP+SURG+AMOX+MET+locally delivered TET. Plus/minus values are mean±standard deviations. There were no significant baseline differences between patients who were in the control group (SRP) and those receiving other treatments as evidenced by the large *p*-values. Box-plots for the mean distribution of the natural log counts of *Actinomyces* and red, orange, yellow, purple, and green complexes are given in Figs. 1A, 2A, 3A, S1A, S2A, and S3A, respectively.

### Model interpretation

We present the incidence rate ratios (IRRs) from the NB2 GEE models in Tables 2–4 and Tables S1–S4. The IRR in part (c) of each table represents the *change* in counts at a certain time point for each treatment group relative to the *change* in the reference group (SRP). Hence, the IRR conveys information on relative differences in changes from 3 months, and *not* on absolute values. If the IRR is significantly less than 1, then the treatment group has experienced a relative decrease of bacterial counts compared with the development in the reference group, SRP. Conversely, if the IRR is significantly larger than 1, then the treatment group has experienced a relative increase of bacterial counts relative to the development in the reference group. In the case of the red and orange complexes, the treatment is considered to be effective compared with SRP when the IRR is significantly less than 1. In contrast, higher counts of *Actinomyces*, which is associated with periodontal health, are desirable. In this case, the treatment is considered effective compared with SRP when the IRR is significantly more than 1. For example, in Table 2 *Actinomyces* counts were 1.28 times higher at 6 months in subjects who were treated with AMOX+MET+TET compared to subjects who were treated with SRP. The other explanatory variables in Table S4 are interpreted similarly.

**Table 2:** IRR† estimates and confidence intervals for *Actinomyces* counts after adjusting for baseline counts in sites that had an initial PD≥5 mm from the GEE NB model using the exchangeable correlation structure in Stata

		Part (c) Comparison made to counts after 3 months			
		
	(b) IRR: 3 months	6 months	12 months	18 months	24 months
	
Treatment effect on *Actinomyces* counts	Marginal effects estimate 99% CI	IRR estimate 99% CI	IRR estimate 99% CI	IRR estimate 99% CI	IRR estimate 99% CI
(a) Reference treatment: SRP.		1.48 (0.69, 3.15)	1.43 (0.74, 2.77)	1.42 (0.54, 3.78)	1.06 (0.44, 2.54)
					
Compared with SRP					
SURG+AMOX+MET	0.83 (0.39, 1.78)	1.03 (0.44, 2.44)	1.44 (0.64, 3.19)	1.26 (0.44, 3.60)	1.69 (0.65, 4.39)
SURG+TET	1.09 (0.51, 2.35)	0.95 (0.40, 2.23)	1.19 (0.53, 2.66)	1.07 (0.37, 3.06)	1.45 (0.54, 3.86)
SURG‡	0.84 (0.40, 1.77)	1.37 (0.60, 3.12)	1.50 (0.71, 3.20)	1.06 (0.38, 2.95)	1.53 (0.58, 3.99)
AMOX+MET+TET	0.83 (0.39, 1.77)	1.28 (0.55, 2.95)	1.34 (0.63, 2.86)	1.40 (0.49, 4.03)	0.96 (0.37, 2.46)
AMOX+MET	1.15 (0.55, 2.42)	0.90 (0.39, 2.06)	0.90 (0.39, 2.07)	0.91 (0.31, 2.64)	1.09 (0.39, 3.03)
TET	0.94 (0.42, 2.09)	1.05 (0.45, 2.46)	0.91 (0.40, 2.05)	0.86 (0.29, 2.53)	1.31 (0.46, 3.70)
SURG+AMOX+MET+TET	1.35 (0.62, 2.92)	0.93 (0.41, 2.09)	1.00 (0.48, 2.11)	0.86 (0.30, 2.45)	1.18 (0.45, 3.11)

There were no significant changes in counts of *Actinomyces* that were observed in all treatment groups compared to counts in the reference group, SRP.

† Incidence rate ratio=exp (*β*).

‡ SURG was performed 3 months after the baseline visit. Results shown at the 3-month study period were for SRP.

SURG=surgery; AMOX=amoxicillin; MET=metronidazole; TET=tetracycline; IRR=incidence rate ratios.

To assist in the interpretation of the results, part (a) in each table reports changes in counts from 3 months for the reference group (SRP) after adjusting for baseline counts. For example, in Table 3, counts of the red complex significantly increased by 77% after 24 months. Part (b) shows the IRR for changes in counts at 3 months from baseline compared to the reference group, SRP. For example, in Table 3 changes from baseline were significantly lower by 37% in subjects who were treated with AMOX+MET+TET compared to subjects treated with SRP.

**Table 3:** IRR† estimates and confidence intervals for red complex counts after adjusting for baseline counts in sites that had an initial PD ≥5 mm from the GEE NB model using the exchangeable correlation structure in Stata

		Part (c) Comparison made to counts after 3 months			
		
	(b) IRR: 3 months	6 months	12 months	18 months	24 months
	
Treatment effect on red complex counts	Marginal effects estimate 99% CI	IRR estimate 99% CI	IRR estimate 99% CI	IRR estimate 99% CI	IRR estimate 99% CI
(a) Reference treatment: SRP		1.30 (0.98, 1.71)	1.37 (0.99, 1.90)	1.36 (0.96, 1.93)	1.77 (1.13, 2.78)**
					
Compared with SRP					
SURG+AMOX+MET	1.05 (0.61, 1.80)	0.96 (0.58, 1.62)	1.08 (0.61, 1.94)	0.81 (0.47, 1.41)	0.52 (0.24, 1.12)
SURG+TET	0.95 (0.71, 1.29)	0.92 (0.57, 1.48)	0.87 (0.50, 1.53)	0.96 (0.54, 1.69)	0.72 (0.40, 1.32)
SURG‡	1.06 (0.65, 1.70)	1.01 (0.59, 1.73)	0.79 (0.36, 1.75)	0.96 (0.49, 1.90)	0.88 (0.41, 1.90)
AMOX+MET+TET	0.63 (0.43, 0.92)**	0.97 (0.62, 1,52)	1.43 (0.84, 2.43)	1.81 (1.07, 3.06)**	1.23 (0.58, 2.60)
AMOX+MET	0.75 (0.50, 1.14)	0.98 (0.59, 1.64)	1.29 (0.56, 2.95)	1.08 (0.54, 2.15)	1.14 (0.51, 2.52)
TET	0.96 (0.68, 1.35)	0.85 (0.47, 1.53)	1.08 (0.61, 1.91)	1.19 (0.62, 2.30)	1.01 (0.50, 2.04)
SURG+AMOX+MET+TET	0.91 (0.60, 1.39)	0.78 (0.43, 1.40)	0.99 (0.56, 1.78)	0.89 (0.46, 1.75)	0.74 (0.34, 1.63)

We observed a 3-month significant reduction in changes of the red complex counts from baseline of 37% in subjects treated with AMOX+MET+TET compared to SRP-treated subjects. No other significant reductions of the red complex counts in sites with severe chronic periodontitis were observed from all study treatments compared to SRP-treated subjects.

† Incidence rate ratio=exp (*β*).

‡ SURG was performed 3 months after the baseline visit. Results shown at the 3-month study period were for SRP.

** Significant results at *α*=0.01.

SURG=surgery; AMOX=amoxicillin; MET=metronidazole; TET=tetracycline; IRR=incidence rate ratios.

Finally, we tested the joint significance of treatment and time (and the interaction) to see whether there were any treatment effects. For the analysis of counts, we found that the overall tests were significant (*p*<0.01). Because of these differences, we shall proceed and report below the developments in treatment effects that were observed over time.

### Treatment effects over time

From the computed IRR and their confidence intervals for sites with severe chronic periodontitis in Table 2, we did not observe significant changes in counts of *Actinomyces*. Figure 1 shows the median distribution of *Actinomyces* counts in box-plots at baseline. In all treatment groups, there was very little variation in the median distribution of *Actinomyces* counts at baseline. Figure 2 shows changes in counts of *Actinomyces* from baseline for each treatment group. For example, there was a sharp decrease in *Actinomyces* counts between baseline and 3 months in the AMOX+MET+TET and TET treatment groups.

**Fig. 4:**
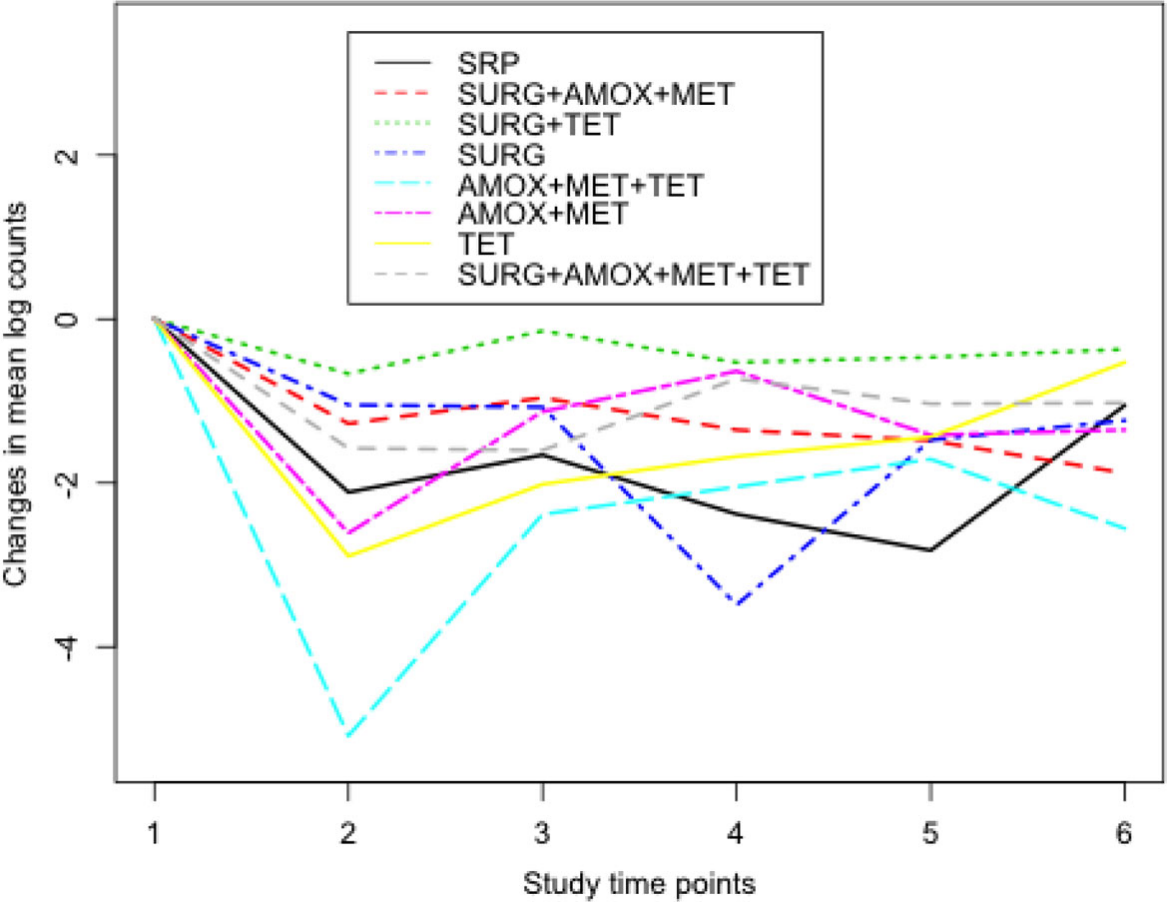
Changes in natural log counts of the red complex at each study time point from baseline. Study time points 1–6 correspond to baseline, 3, 6, 12, 18, and 24 months, respectively. Much lesser counts of the red complex were observed between baseline and 3 months in subjects who were treated with AMOX+MET+TET.

The median distribution of the red complex counts at baseline is given in Fig. 3. The IRR for the red complex counts in sites that had an initial PD of at least 5 mm and had been treated with AMOX+MET+TET was 0.63 after 3 months as shown in Table 3 (see also Fig. 4). All other things being equal and compared to SRP, this indicates that subjects who were treated with AMOX+MET+TET had changes in counts of the red complex significantly reduced from baseline by 37% after 3 months. Significant changes in counts of *T. forsythia* of the red complex were also observed at 3 months when AMOX+MET+TET and AMOX+MET were used compared to *T. forsythia* counts in the reference group SRP at baseline. The two treatments had similar short-term beneficial changes in the composition of *T. denticola*, whereas SURG had a short-term effect on *P. gingivalis* (not presented).

**Fig. 5:**
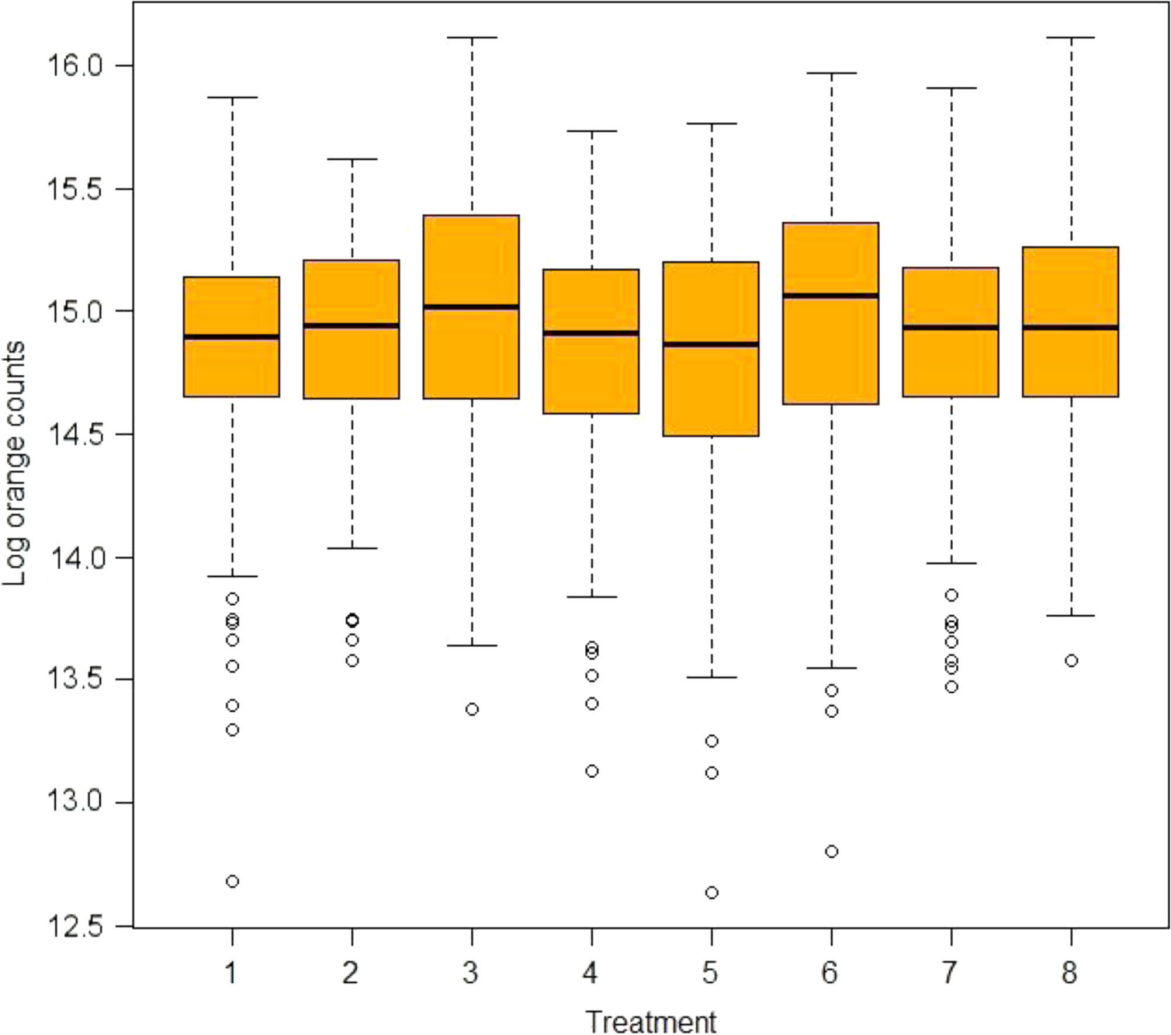
Box-plot showing the median distribution of orange complex counts by treatment at baseline. The treatments 1–8 are SRP, surgery (SURG)+AMOX+MET, SURG+TET, SURG, AMOX+MET+TET, AMOX+MET, TET, and SURG+AMOX+MET+TET, respectively. Again, minimal variations in the median distributions of the counts can be seen.

**Fig. 6:**
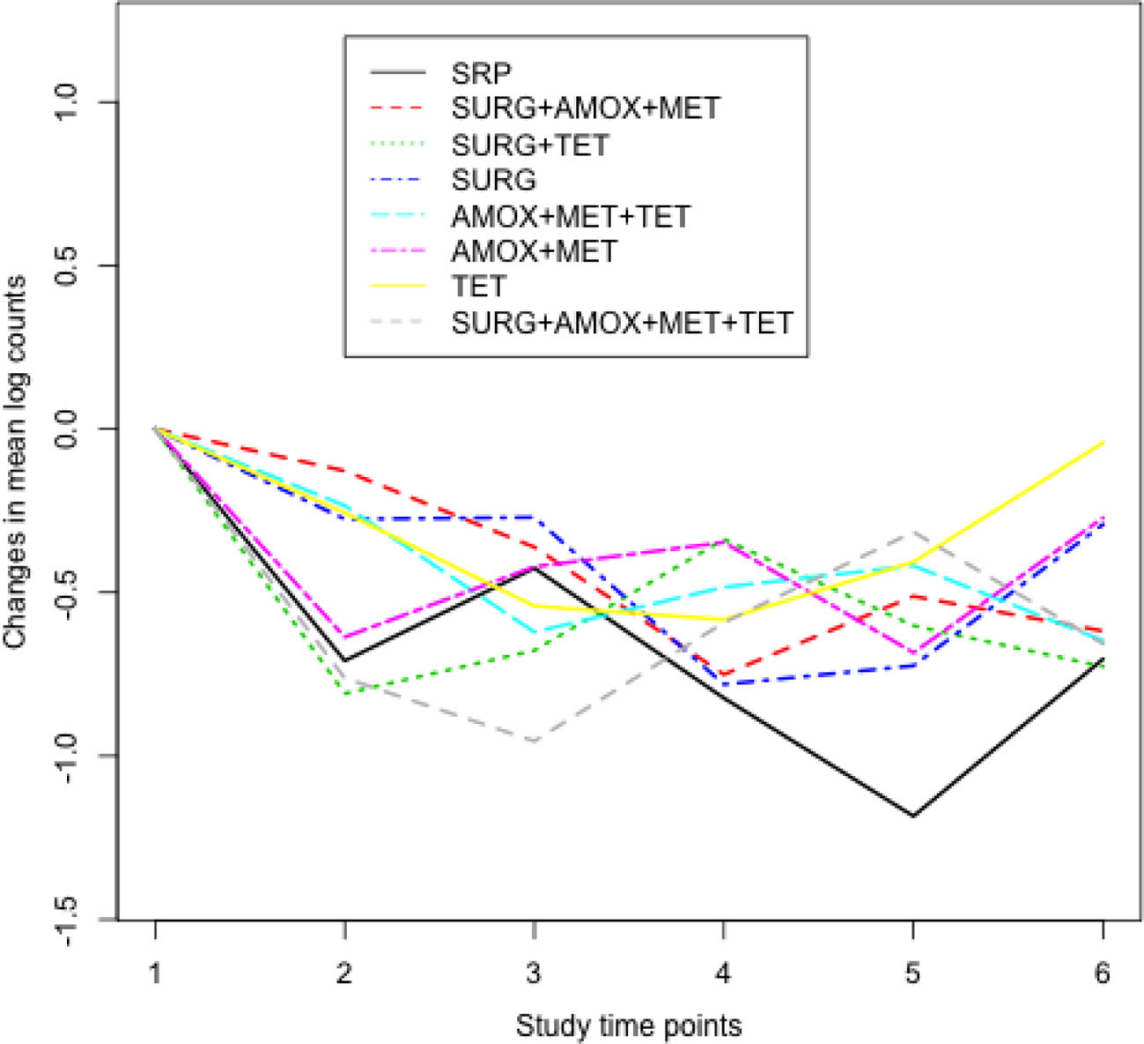
Changes in natural log counts of the orange complex at each study time point from baseline. Study time points 1–6 correspond to baseline, 3, 6, 12, 18, and 24 months, respectively. Subjects treated with surgery (SURG)+TET had lesser counts at 3 months.

Figure 5 shows that there were minimal variations in the median distributions of the orange complex counts at baseline while Fig. 6 shows that compared to SRP, counts in the other treatment groups were higher between 12 and 24 months. However, there were no significant beneficial changes in counts of the orange complex that were observed over time as shown in Table 4.

**Table 4:** IRR† estimates and confidence intervals for orange complex counts after adjusting for baseline counts in sites that had an initial PD ≥5 mm from the GEE NB model using the exchangeable correlation structure in Stata

		Part (c) Comparison made to counts after 3 months			
		
	(b) IRR: 3 months	6 months	12 months	18 months	24 months
	
Treatment effect on orange complex counts	Marginal effects estimate 99% CI	IRR estimate 99% CI	IRR estimate 99% CI	IRR estimate 99% CI	IRR estimate 99% CI
(a) Reference treatment: SRP					
		0.90 (0.68, 1.18)	0.89 (0.67, 1.19)	0.82 (0.66, 1.02)*	1.02 (0.75, 1.37)
					
Compared with SRP					
SURG+AMOX+MET	0.90 (0.68, 1.18)	1.04 (0.74, 1.46)	1.09 (0.76, 1.58)	1.39 (1.04, 1.88)**	0.97 (0.68, 1.39)
SURG+TET	0.92 (0.70, 1.21)	1.14 (0.77, 1.70)	1.18 (0.82, 1.70)	1.36 (0.97, 1.91)*	1.05 (0.71, 1.56)
SURG‡	0.88 (0.62, 1.24)	1.20 (0.76, 1.89)	1.08 (0.71, 1.64)	1.39 (0.90, 2.13)*	1.17 (0.77, 1.77)
AMOX+MET+TET	1.01 (0.75, 1.38)	0.95 (0.65, 1.41)	1.01 (0.65, 1.59)	1.19 (0.86, 1.65)	1.02 (0.68, 1.53)
AMOX+MET	0.89 (0.66, 1.20)	1.17 (0.79, 1.73)	1.14 (0.76, 1.70)	1.27 (0.90, 1.78)	1.09 (0.73, 1.63)
TET	1.05 (0.76, 1.44)	1.01 (0.63, 1.62)	0.97 (0.66, 1.43)	1.02 (0.71, 1.46)	0.95 (0.62, 1.45)
SURG+AMOX+MET+TET	0.94 (0.70, 1.25)	0.94 (0.67, 1.31)	1.01 (0.71, 1.44)	1.25 (0.86, 1.81)	0.98 (0.68, 1.41)

There were no significant reductions in orange complex counts that were observed in all treatment groups compared to SRP.

† Incidence rate ratio=exp (*β*).

‡ SURG was performed 3 months after the baseline visit. Results shown at the 3-month study period were for SRP.

* Significant results at *α*=0.05.

** Significant results at *α*=0.01.

SURG=surgery; AMOX=amoxicillin; MET=metronidazole; TET=tetracycline; IRR=incidence rate ratios.

In Fig. S1, higher counts of the yellow complex were observed in the SURG, SURG+AMOX+MET, and SURG+TET groups compared to the reference, SRP. However, as shown in Fig. S1A, the three treatment groups had lesser counts of the yellow complex at baseline. At the 5% significance level, sites that were treated with SURG+AMOX+MET had an increase in counts of the yellow complex of 85% and 70% after 18 and 24 months, respectively, compared to SRP-treated subjects as shown in Table S1.

There were minimal variations in the median distributions of the purple complex counts at baseline as shown in the box-plots of Fig. S2A. As shown in Fig S2, decreases in counts of the purple complex were observed between baseline and 3 months in all treatments except in the SURG-treated group. However, there were no significant changes in counts of the purple complex that were observed in all treatment groups as shown in Table S2. Similar to the box-plots of Fig. S2A, minimal variations in the median distributions of the green complex counts were also observed as shown in Fig. S3A. Figure S3 shows that compared to SRP, much lesser counts of the green complex were observed at 3 months in the TET and AMOX+MET+TET-treated groups. As demonstrated in Table S3, we observed a 42% and 50% decrease in counts of the green complex after 6 and 12 months, respectively, in subjects treated with SURG. Actually, all treatments with SURG reduced green counts at 12 months as shown in Table S3.

### Other predictors

We also tested the effects of gender, smoking habits, nationality, age, PD, accumulation of plaque, BOP, gingival redness, and CAL as predictors of counts of the complexes over time.

We did not observe significant differences in counts of the complexes and *Actinomyces* between males and females in sites that had an initial PD of at least 5 mm and then treated, as shown in Table S4. Red and orange complexes’ counts significantly increased in smokers by 18% and 8%, respectively. However, smoking significantly reduced counts of *Actinomyces* by 10%. Count levels of the orange and green complexes were significantly lower in Swedish subjects while *Actinomyces* counts were significantly higher compared to American subjects. Further increase in PD significantly elevated counts of the red and orange complexes by 6% and 3% respectively. Counts of the red complex were also higher in sites that were bleeding, lost more attachment, and had gingival redness.

### Fractional responses

As with count models, we tested the joint significance of treatment and time (and the interaction) to see the extent in which the treatments influenced the composition of the bacteria. Although the overall tests were not significant, we observed findings that were comparatively similar to the count responses. For example, in Table S5 (baseline covariates not presented), we observed a short-term benefit of using AMOX+MET+TET. However, just like the analysis of the count data, there were no significant benefits that were observed with the other treatments. The analysis of proportions also showed that smoking increased the odds of the red complex by 22%, deeper pockets increased the odds of the red complex by 7%, the odds of the red complex was 4% higher in subjects who had experienced further losses in clinical attachment, and gingival redness and BOP increased the odds of the red complex by 10% and 8%, respectively. In the analysis of red counts (Table 3), smoking, deeper pockets, further losses in attachment, gingival redness, and BOP elevated the red counts by 18%, 6%, 3%, 12%, and 10%, respectively.

## Discussion

Knowledge suggesting that periodontal pathogens operate in complexes rather than as single pathogens has led to changes in treatment approaches to be considered. This study examined over 2 years the effect of different periodontal therapies, including combinations of therapies on different bacterial species of the subgingival microbiota present in five different complexes (2) with the aim of selecting the most beneficial periodontal therapy. We specifically looked at changes that occur in severely affected mesiobuccal sites (PD≥5 mm). Taking samples from all sites might have given a better view of the total cultivable microbiota. However, we know from previous work that the red and orange complexes are the ones most related to clinical disease, particularly in terms of PD. Therefore, we felt that selecting mesiobuccal sites would give a good overall insight into what happened with the agents most related to periodontitis as a result of local/systemic antibiotic treatment. Treatment effects on single species of the red complex were also examined.

Although short-term improvements in the counts of the red complex were observed in sites that were treated with AMOX+MET+TET, long-term significant effects were not observed with any of the eight study treatments. We also examined the effect of BOP, accumulation of plaque, deeper pockets, and smoking on counts of the complexes. We believe that these four factors were most important for diminishing the effects of the study treatments. For example, about 41% of our study subjects were current smokers and our study revealed that colonization of sites by periodontal pathogens was more extensive in smokers than in non-smokers. This may explain why smokers are less responsive to periodontal therapies than non-smokers. In a study by Bagaitkar et al. (32) on tobacco-induced alterations to *P. gingivalis*–host interactions, the authors concluded that *P. gingivalis* adapts and changes its DNA and membrane proteins in response to cigarette smoke. This might be one reason why smokers are more likely to be resistant to periodontal treatment and are more susceptible to oral disease caused by infection with *P. gingivalis*. Secondly, there was evidence of poor oral hygiene persisting among our study subjects. For example, SRP was performed in all study subjects at baseline and yet after only 3 months, 20% of the sites had already accumulated dental plaque and 21% of the sites bled on probing. Similar problems were also observed at other study time points implying that the use of either single therapy such as TET or combinational therapies such as AMOX+MET had little impact on the counts. Locally delivered TET has the added advantage of being site-specific. As noted by Pavia et al. (33), local delivery systems are capable of producing high local concentration of agents with very low systemic spillover. However, the study revealed that the use of TET in severely affected sites of subjects who also showed evidence of poor oral hygiene gave no significant improvements in the counts of either the red or the orange complex.

In chronic periodontitis, we desire treatments that elevate *Actinomyces* counts because maintaining a bacterial flora associated with health after treatment is crucial for the prognosis. However, we did not find evidence that the periodontal treatments promoted growth of *Actinomyces* in severely affected sites. Although SURG has long been suspected of initiating loss of CAL through a detrimental effect on osteoblasts, it remains a useful procedure for effective removal of plaque and elimination of periodontal pockets. However, the problems that were observed and discussed above could have also played a major role in diminishing the effectiveness of SURG.

Normally, the Poisson distribution is recommended for analyzing count data. However, due to extra variability in our bacterial data, a suitable model for overcoming the problem of over-dispersion and correlated observations was the negative binomial generalized estimating equation (NB GEE) model. The advantage of the GEE approach is that it gives consistent parameter estimates for correctly specified mean structure even if the working correlation matrix has been misspecified and consistent standard errors can be obtained by using a robust sandwich estimator. A major limitation of our analysis using the GEE approach is that it is generally restricted to one level of correlation. Whereas mixed models can fit multiple levels of correlations, for example teeth nested in subjects, with the GEE approach our analysis was restricted to ‘within subject’ dependencies only. Alternative approaches include using random effects negative binomial (RENB) and/or the zero-inflated models with robust standard errors.

An attempt was also made in this study to analyze proportions of counts and compare these findings to the results from the analyses of counts. By using proportions, information about absolute changes in the levels of the species being investigated is usually lost. The analysis of proportions in this study aimed at investigating the effect of the interventions compared to SRP on changes in the composition of the complexes. However, the findings were comparatively similar and we did not find any noteworthy long-term treatment effect.

Whereas in our previous study (1) SURG+AMOX+MET+TET gave the best maintenance of clinical results over a 2-year period, long-term significant microbiological effects were not obtained with any of the treatments, although sites treated with AMOX+MET+TET showed clear reductions in the counts of the red complex after 3 months. The reason for this is not clear. Possibly, unfavorable post-treatment ecological changes require some time to develop before re-infection is established. This supports the notion that we have no concept of the length of time that separates changes in the subgingival microbiota and periodontal tissue destruction (34). Also, initiation of disease does not necessarily coincide with the detection of disease since there may be a latent period between them (35).

In conclusion, short-term reduction in counts of the red complex in deeper sites were observed with AMOX+MET+TET. However, the treatments did not produce significant beneficial changes in counts of the orange complex and *Actinomyces* in these sites. After treatment red and orange complex counts significantly increased in smokers, and smoking significantly reduced counts of *Actinomyces*. Count levels of the orange and green complexes were significantly lower in Swedish subjects, while *Actinomyces* counts were significantly higher compared to American subjects. Further increase in PD significantly elevated counts of the red and orange complexes. Counts of the red complex were also higher in sites that were bleeding, lost more attachment, and had gingival redness.

We found that BOP, accumulation of plaque, deeper pockets, and smoking had detrimental effects on the counts of the complexes and believe that these four factors were mainly responsible for diminishing the effects of the study treatments. Our study clearly showed that antibiotic-treated patients tend to suffer relapse of the microbiota associated with periodontitis unless dental plaque is prevented from re-accumulating either by self-inflicted personal oral hygiene or professional maintenance therapy. The importance of monitoring risk predictors during maintenance therapy such as smoking and probing depth was also indicated.
